# The LIFE-MET trial: effect of insulin sensitization on pubertal progression following lifestyle intervention and/or treatment with metformin in girls with early puberty and overweight: study protocol for a randomized, placebo-controlled trial

**DOI:** 10.1186/s13063-025-09229-3

**Published:** 2025-11-25

**Authors:** Grith Laerkholm, Line Anker Bang Thybo, Astrid Bruun Rasmussen, Ann-Margrethe Rønholt Christensen, Annette Korsholm Mouritsen, Julie Tonsgaard Kloppenborg, Jesper Johannesen, Ajay Thankamony, Ken K. Ong, Lasse Gliemann, Rikke Beck Jensen

**Affiliations:** 1 Department of Pediatrics and Adolescent Medicine, Herlev and Gentofte Hospital, Copenhagen University Hospital, Copenhagen, Denmark; 2 Department of Pediatrics, Kolding Hospital, University Hospital Lillebaelt, Vejle, Denmark; 3 Department of Pediatrics and Adolescent Medicine, Aarhus University Hospital, Aarhus, Denmark; 4 Department of Pediatrics, Aalborg University Hospital, Aalborg, Denmark; 5 Department of Pediatrics, Zealand University Hospital Roskilde, Roskilde, Denmark; 6 Steno Diabetes Center Copenhagen, Copenhagen, Denmark; 7 Institute for Clinical Medicine, University of Copenhagen, Copenhagen, Denmark; 8 Cambridge University Hospital NHS Trust, Cambridge, UK; 9 MRC Epidemiology Unit, Institute of Metabolic Science, University of Cambridge, Cambridge, UK; 10 Department of Nutrition and Exercise, Copenhagen University, Copenhagen, Denmark

**Keywords:** Early puberty, Overweight, Adiposity, Insulin resistance, Lifestyle, Metformin

## Abstract

**Background:**

Puberty in girls is occurring earlier worldwide with a declining trend over the recent decades, resulting in increased attention on the accompanying risks of psychosocial challenges and adverse health outcomes for the affected girls. Diverse mechanisms have been proposed as mediators of the tendency for earlier pubertal maturation, including a shift toward a more sedentary lifestyle and changes in dietary habits leading to childhood obesity. Several studies have demonstrated a potential association between the rise in childhood obesity prevalence and the decline in the age at which puberty begins. Increased insulin resistance is thought to play a role in this connection, and previous studies indicated that improved insulin sensitivity following either treatment with metformin or weight loss could delay pubertal progression in girls with overweight.

**Methods:**

LIFE-MET is a randomized, placebo-controlled, four-arm, multicenter trial of girls (*n* = 80) with overweight and early puberty. Allocation to metformin or placebo will be double-blinded. Eligible girls will be randomly assigned to one of the four study arms: Metformin + lifestyle intervention (*N* = 20), Metformin alone (*N* = 20), Placebo + lifestyle intervention (*N* = 20), Placebo alone (*N* = 20). The intervention period is 6 months with a follow-up after an additional 6 months. The primary outcome is the change in bone age from baseline to 12 months, as a marker of pubertal progression. Secondary outcomes include changes in body composition, Tanner stage, fitness level, sex hormones, insulin resistance, and age at menarche.

**Discussion:**

New strategies not only for the treatment but also for the prevention of both overweight and early puberty are needed. The LIFE-MET trial is a randomized controlled trial with a combination of lifestyle intervention including online, virtual reality training and a pharmacological intervention consisting of metformin and/or placebo, to our knowledge the first of its kind. We expect that this intervention will have a beneficial effect on both pubertal progression, daily physical activity level, and body composition. Additionally, a healthier body composition may have beneficial effects on long-term co-morbidities related to early puberty.

**Trial registration:**

Clinical Trial Information System (CTIS): 2024-511009-50-00. Approved 04/30/2024. https://euclinicaltrials.eu/search-for-clinical-trials/?lang=en&EUCT=2024-511009-50-00.

## Administrative information

NOTE: the numbers in curly brackets refer to SPIRIT checklist item numbers [1]. The order of the items has been modified to group similar items (see http://www.equator-network.org/reporting-guidelines/spirit-2013-statement-defining-standard-protocol-items-for-clinical-trials/).

**Table:** 

Title {1}	The LIFE-MET Trial: Effect of insulin sensitization on pubertal progression following lifestyle intervention and/or treatment with Metformin in girls with early puberty and overweight: A randomized, placebo-controlled trial.
Trial registration {2a} and {2b}	EU-CT number: 2024-511009-50-00 [Clinical Trial Information System (CTIS)]. Approved 04/30/2024. https://euclinicaltrials.eu/search-for-clinical-trials/?lang=en&EUCT=2024-511009-50-00 NCT05957991 [Clinical Trials; clinicaltrials.gov]. Registered 07/14/2023.
Protocol version {3}	1.3, 17/10/2024
Funding{4}	The DAnish Precocious Puberty (DAPP) study is funded by a Non-Diabetic Endocrinology Collaborative grant of 9.9 million DKK from the Novo Nordisk Foundation in January 2022. This grant will support the initiation of the LIFE-MET Trial. The foundation “Læge Sofus Carl Emil Friis og Hustru Olga Doris Friis’ Legat” has supported LIFE-MET with 421.600 DKK. The foundation “Aase og Ejnar Danielsens Fond” has supported LIFE-MET with 200.000 DKK. Further granting has been and will be applied for.
Author details {5a}	1) Department of Pediatrics and Adolescent Medicine, Herlev and Gentofte Hospital, Copenhagen University Hospital. 2) Department of Pediatrics, Kolding Hospital, University Hospital Lillebaelt 3) Department of Pediatrics and Adolescent Medicine, Aarhus University Hospital 4) Department of Pediatrics, Aalborg University Hospital 5) Department of Pediatrics, Zealand University Hospital Roskilde 6) Steno Diabetes Center Copenhagen 7) Institute for clinical medicine, University of Copenhagen 8) Cambridge University Hospital NHS Trust, Cambridge, UK 9) MRC Epidemiology Unit, Institute of Metabolic Science, University of Cambridge, Cambridge, UK 10) Department of Nutrition and Exercise, Copenhagen University
Name and contact information for the trial sponsor {5b}	Investigator initiated clinical trial; RB Jensen (Principal Investigator); rikke.beck.jensen@regionh.dk
Role of sponsor {5c}	This is an investigator initiated clinical trial. The funders played or will play no role in neither the design of the trial, the collection, analysis, and interpretation of data, or in writing the manuscript.
Composition, roles, and re-sponsibilities of the coordina-ting centre, …, and other individuals or groups overseeing the trial {5d}	The coordinating centre (Department of Pediatrics and Adolescent Medicine, Herlev and Gentofte Hospital) is composed by PI and trial sponsor RBJ and daily coordinating investigator GL. The local nurses and PI’s at each center will provide day-to-day support for the trial. Regular meetings between PI’s will take place to coordinate and supervise the tasks. The trial will be monitored by the Good Clinical Practice (GCP) Units. See {21a} for data monitoring committee.

## Introduction

### Background and rationale {6a}

Puberty in girls is occurring earlier worldwide with a descending trend over the recent decades [2–8]. The age of breast development has declined most markedly [3, 5, 6] but several studies have also demonstrated an earlier age at menarche, although to a lesser extent [6, 9–13]. The onset of puberty is strongly influenced by genetic factors; however, genetics cannot account for the significant changes observed in the changes in age at pubertal onset. Diverse mechanisms have been proposed as mediators of earlier pubertal maturation. These include improvements in health and nutrition [14, 15], as well as lifestyle factors such as sedentary behavior and dietary practices that contribute to childhood obesity [2]. Early puberty is associated with several adverse health outcomes, but short-term consequences are primarily adverse mental health outcomes such as depression, anxiety, and eating disorders, as well as increased risk-taking behavior and earlier onset of sexual activity [16–18]. Long-term health challenges include an increased risk of type 2 diabetes, cardiovascular disease, and breast cancer [16, 19–22]. Taken together, this necessitates continued attention on understanding the underlying mechanisms affecting the initiation of puberty.

### Increasing incidence of childhood overweight and relation to early puberty

For several decades, the prevalence of childhood obesity has escalated globally and remains high [23–26]. According to the World Health Organization, 29% of European children aged 7–9 years were overweight during the period 2018–2020 [26].

Recent epidemiological studies have demonstrated a potential association between the rise in obesity prevalence and the decline in the age at which breast development begins [27, 28]. However, this relationship does not confirm obesity as a causal factor of earlier puberty, and this remains a subject of debate. Several mechanisms have been proposed, including increased insulin resistance [29].

Children with obesity often exhibit increased levels of androgens from both the adrenal glands and ovaries, as a response to the increased insulin resistance and compensatory hyperinsulinemia [24, 30]. Insulin resistance is also associated with decreased concentrations of sex hormone binding globulin (SHBG), resulting in an increased availability of sex steroids [29]. Evidence suggests that not only adiposity but also the “mismatch” between low birth weight and rapid weight gain in early infancy may influence pubertal timing, likely due to increased production of adrenal androgens and decreased insulin sensitivity [24, 30–32].

Normal puberty is a period characterized by insulin resistance [33, 34], and a recent systematic review and meta-analysis demonstrated an independent association between early age at menarche and insulin resistance, also when adjusting for total body fat percentage [35–37]. The causal relationship is still uncertain, but supporting the role of insulin resistance in early menarche are the clinical studies of Ibañez et al. Focusing on girls born small for gestational age (SGA) experiencing early puberty, they revealed that treatment with metformin, which enhances insulin sensitivity, delayed pubertal progression and age at menarche by nearly 1 year compared to the untreated group [38]. In another study of girls with low birth weight and precocious pubarche, metformin lowered androgen levels and delayed the clinical onset of puberty by 0.4 year [39]. Additionally, a clinical study of children with overweight participating in a 1-year weight loss program including lifestyle interventions demonstrated that weight loss was associated with a significant later onset of puberty in girls [40]. Thus, there is some evidence that improved insulin sensitivity obtained either by treatment with metformin or following weight loss could delay pubertal progression in girls.

### Physical activity and timing of puberty

Increased levels of physical activity have been linked to later pubertal maturation, the most pronounced association being for professional athletes [41, 42]. A randomized controlled trial (RCT) examining the impact of combined lifestyle changes (diet, decreased television viewing, and PA) on the timing of puberty demonstrated a 25% lower relative risk of reaching menarche during the study period for the girls in the intervention schools compared to control schools [42]. The effects of increased PA on the progression of puberty have been proposed to be mediated by changes in body composition, in particular adipose tissue and thereby leptin secretion [43], and improved insulin sensitivity. Overall, the evidence of an association between PA and age at menarche remains sparse, as most former studies were historical cohorts or case-control studies [41].

Searching for innovative methods to make children and adolescents more physically active, the effect of exergames or active video games (AVGs) on PA level and BMI reduction has been examined in several RCT studies [44–49]. Trost et al. [50] found that incorporation of AVGs into a pediatric weight management program had positive effects on physical activity and relative weight. Another study concluded that exergaming at home lowered BMI z-score and physical activity levels in children aged 10–12 years with overweight or obesity and proposed that exergaming with social support could be promoted as an exercise option for children [44]. Accordingly, AVGs could be considered a possible supplement in the management of overweight and sedentary lifestyle in children and adolescents.

### Perspectives and aim of this trial

The overall aim of this trial is to explore whether a novel alternative treatment strategy for girls with overweight and early puberty can postpone pubertal maturation. Our hypothesis is that lifestyle factors and adiposity influence pubertal onset and that this process may be reversible through improvement of insulin sensitivity and/or weight loss or changes in body composition.

### Explanation for choice of comparators {6b}

Our protocol consists of a lifestyle intervention (online training and dietary counseling) and a pharmacological intervention with metformin or placebo. We expect both interventions to postpone further pubertal progression by improving insulin sensitivity and/or by changing body composition in girls with overweight and early puberty. Since pharmacological suppression of puberty is not recommended for girls older than 8 years presenting with early puberty, the comparator will be placebo and no lifestyle intervention.

### Objectives {7}

Primary objective: To determine the effects of metformin treatment vs placebo during a 6-month period on bone age as a marker of pubertal maturation in girls with overweight and early puberty.

Secondary objectives:

1. To determine the effects of metformin vs placebo and/or lifestyle intervention on bone age, breast development, height velocity, sex hormones, insulin sensitivity, metabolic markers, blood pressure, fitness level, daily physical activity level, and body composition*.*
2. To determine the effects of metformin vs placebo and/or lifestyle intervention on quality of life (QoL).
3. To determine the effects of metformin vs placebo and/or lifestyle intervention on age at menarche.

### Trial design {8}

The participants will be recruited from the DAnish Precocious Puberty study (DAPP study, Clinical Trial number NCT05957991) which is a nationwide longitudinal cohort study including children with early puberty to determine the incidence of central precocious puberty and to explore the impact of lifestyle and environmental factors on the timing of puberty. The DAPP study, which was initiated on December 1, 2023, will include all children referred for precocious puberty to each of the 17 pediatric departments in Denmark during a 3-year period.

LIFE-MET is a randomized, placebo-controlled, four-arm, multicenter trial with a 1:1 allocation ratio of a sub-cohort (*n* = 80) of participants from the DAPP study. The trial is a superiority trial comparing metformin to placebo. Allocation to metformin or placebo will be double-blinded. Eligible girls will be randomly assigned to one of the four trial arms: Metformin + lifestyle intervention (*N* = 20), Metformin alone (*N* = 20), Placebo + lifestyle intervention (*N* = 20), Placebo alone (*N* = 20). The intervention period is 6 months with follow-up after an additional 6 months.

## Methods: participants, interventions and outcomes

### Study setting {9}

The trial subjects will be recruited from five pediatric departments, each located in one of the five regions in Denmark: (1) Copenhagen University Hospital—Herlev (Capital Region), (2) Zealand University Hospital—Roskilde (Region Zealand), (3) Lillebaelt University Hospital—Kolding (Region of Southern Denmark), (4) Aarhus University Hospital (Central Denmark Region), (5) Aalborg University Hospital, Nord (North Denmark region). All trial centers provide an appropriate setting to examine participants and conduct study visits including the necessary equipment to perform the trial.

### Eligibility criteria {10}

Eligible participants are girls (*n* = 80), aged 8.0–9.5 years from the DAPP cohort with clinical and biochemical signs of early puberty and overweight.

#### Inclusion criteria

- Age 8.0–9.5 years
- Breast development on clinical examination (≥Tanner B2)
- GnRH test with stimulated LH > 5 IU/l or non-stimulated LH > 0.3 IU/L
- Overweight defined by the International Obesity Task Force (IOTF) as BMI > 91 st centile (1.34 SDS) [51]
- Premenarcheal

#### Exclusion criteria

- Known or suspected hypersensitivity or allergy to metformin
- Diabetes mellitus
- Previous or active malignancy
- Cardiac, pulmonary, hepatic, or renal diseases associated with significant decompensation
- Psychological problems likely to lead to significant non-compliance

### Interventions {11}

#### Interventions for each group {11a}

Participating girls will be randomly distributed into four subgroups (1:1):

- 1a. Metformin + lifestyle intervention (*N* = 20)
- 1b. Metformin alone (*N* = 20)
- 2a. Placebo + lifestyle intervention (*N* = 20)
- 2b. Placebo alone (*N* = 20)

##### Medication intervention

Metformin subgroups: Trial dose will be 1000 mg daily (1 scored tablet). Initiation dose will be 500 mg (½ tablet) for the first 14 days to avoid or mitigate adverse effects. The treatment period will last for 6 months in total (180 days).

Placebo subgroups: The placebo dose will be ½ tablet once daily for 14 days, and then 1 tablet once daily for the remaining period. The treatment period will last for 6 months in total (180 days).

The tablets containing the active substance (metformin) and those containing placebo will be indistinguishable from one another with the same shape, size, and color and will both have a score line.

##### Lifestyle intervention

The lifestyle intervention will consist of:

Nutritional counseling for the parents in groups is performed online by a trained physician:

1. The nutritional counseling will consist of an online session after the baseline visit where parents will be informed about the latest recommendations of a healthy diet for children from the Danish Food Agency, including guidance for healthier grocery shopping. In addition, information leaflets developed by the Danish Food Agency will be handed out or send to the parents.

An online training program twice a week for 6 months supervised by training experts with a background in exercise physiology:

2. The online training will consist of both aerobic exercises and strength training. Training sessions will take place online twice a week and will consist of circuit training and active virtual reality gaming (AVRG) using the application FitXR, MetaQuest3. The participants will be able to interact and exercise with a private group with their coach and peers in the application FitXR.

Circuit training is a workout that involves rotating through various exercises targeting different parts of the body, such as squats, burpees, and jumping jacks. It involves endurance training, high-intensity aerobics, and exercises performed in a circuit, similar to high-intensity interval training. Sessions will begin with a warm-up, followed by a 5–20-min circuit training which increases throughout the intervention period following a schedule, and ending with 15–20 min of AVRG using FitXR. The AVRG will primarily consist of dancing or boxing sessions, as these were judged to be the most suitable sports in FitXR for children in this age group during a feasibility trial.

Participants in the lifestyle intervention group will receive a fitness watch (Garmin Vivofit Junior 3) with the purpose of displaying daily steps taken and motivating the girls to increase their daily physical activity level.

Fitness level (aerobic performance and explosive strength) will be evaluated for all participants prior to and after cessation of the intervention period using the following validated tests adapted to children: standing long jump, a 20-m sprint, and the YYIR1C (Yo-Yo Intermittent Recovery Level 1 for Children) [52]. In addition, the participants should wear an activity tracker for 1 week prior to and after cessation of the intervention period.

#### Criteria for discontinuing or modifying allocated interventions {11b}

In case of gastrointestinal adverse effects of the medication, participants can be advised to divide the daily dose over two doses. In all participating girls, safety blood samples will be performed at baseline and at 3 and 6 months including renal (sodium (Na), potassium (K), creatinine, urea) and hepatic function tests (alanine transaminase (ALAT), bilirubin). A progressive increase of creatinine, urea, ALAT, or potassium levels will be a reason for discontinuing the medication. Persistent gastrointestinal adverse effects (vomiting, nausea, abdominal pain) and a persistent skin rash are also reasons for discontinuation of the medication.

Participants will be instructed to pause treatment with metformin or placebo in case of moderate or severe dehydration/repeated vomiting, severe infection requiring hospitalization, or in case of an operation requiring general, spinal, or epidural anesthesia. Participants will also be instructed to inform health personnel of their participation in the trial in case of hospitalization. In case of pausing treatment, participants are asked to inform the local PI.

#### Strategies to improve adherence to interventions protocols {11c}

Participants will be contacted by telephone after 1 month and will be seen every 3 months during the treatment period to maintain close contact and to monitor adherence and adverse events. In addition, participating girls and their families will be asked to fill out a short daily diary including adherence (tablets taken). Ultimately, adherence will be registered when participants return any unused medication after the intervention has ended (ratio between tablets dispensed and tablets returned by the patient at 6 months).

#### Relevant concomitant care permitted or prohibited during the trial {11d}

The participant can continue her concomitant therapy, except for any hormonal treatment including GnRH-agonists, sex hormones, or contraception.

### Provisions for post-trial care {30}

Not applicable. No such provisions have been made.

### Outcomes {12}

Primary outcome: Change in bone age from baseline to 12 months (difference in days).

Secondary outcomes:

- Breast development (puberty Z-scores [53])
- Anthropometry (height, weight, body mass index (BMI), waist-hip circumference, and ratio)
- Body composition (body fat percentage, lean mass, and gynoid/android fat distribution)
- Sex hormones (LH, FSH, estradiol, androgens, SHBG, AMH, and inhibin B)
- Insulin sensitivity (HOMA-IR) (calculated from fasting insulin and glucose)
- Age at menarche (electronic questionnaires)

Exploratory outcomes:

- Systolic blood pressure and resting heart rate
- Metabolic markers (i.e., cholesterol, triglyceride, leptin, and adiponectin)
- Fitness level (score)
- Daily physical activity level (time spent in different activity categories)
- Daily steps taken (only lifestyle intervention groups)
- Assessment of quality of life by online questionnaires (WHO-5 Well Being Score)

Safety parameters:

- Kidney and hepatic function tests (Na, K, urea, creatinine and ALAT, bilirubin)

Change in bone age after 12 months was chosen as the primary endpoint to evaluate the progression of puberty because both clinical and biochemical evaluations of pubertal stage can be challenging to accurately quantify. Automated determination of bone age will give us an objective measure of bone maturation which reflects growth velocity as an indicator of pubertal progression.

### Participant timeline {13}

**Fig. 1:**
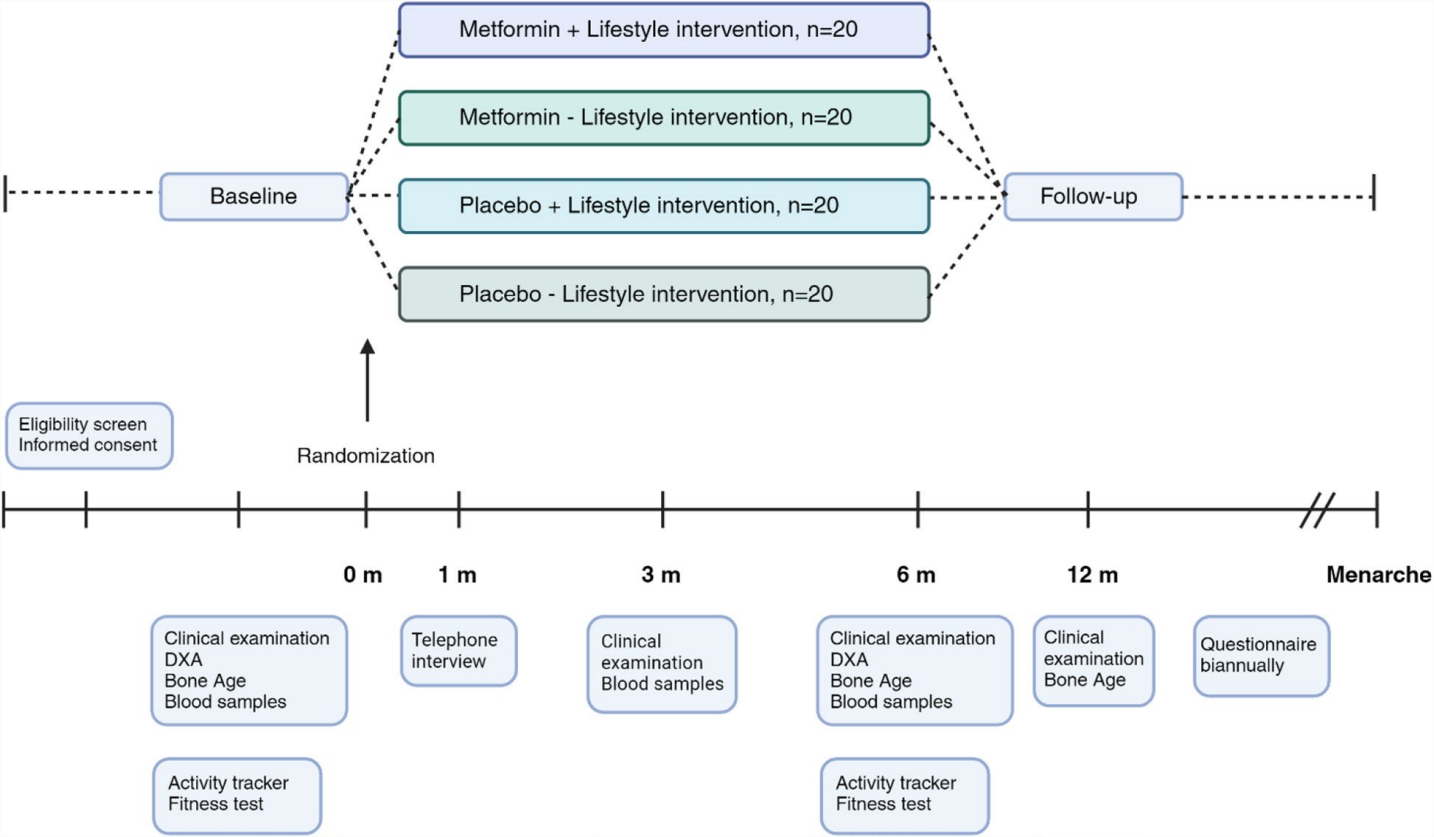
Diagram of the trial design

**Fig. 2:**
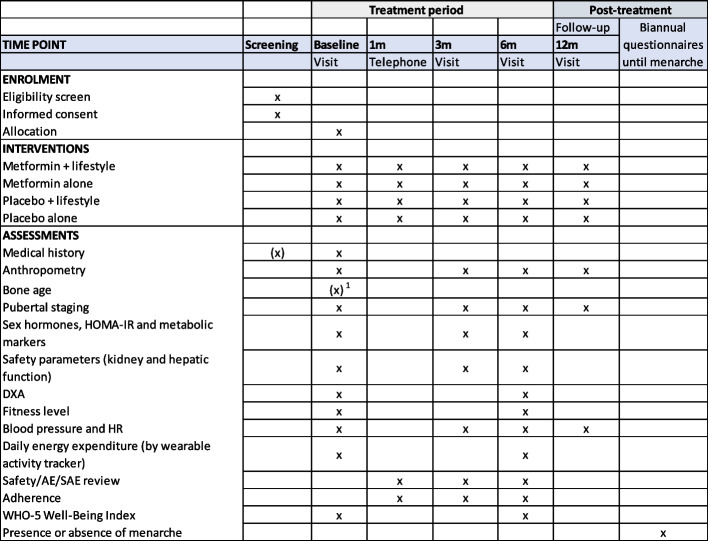
Time schedule of enrollment, interventions, assessments and visits for participants. Cf. the SPIRIT 2013 guideline (1)

Figure 1 outlines the diagram of the trial design. Figure 2 is a time schedule of enrollment, interventions, assessments, and visits for participants cf. the SPIRIT guideline (1).

### Sample size {14}

Sample size was calculated using a study of girls with low birth weight who had early puberty and were treated with metformin. This treatment delayed pubertal progression, and age at menarche was 11.4 (SD 0.69) years in untreated girls and 12.5 (SD 0.44) years in metformin-treated girls (*P* < 0.0001) [38]. This difference is equal to 1 SD of age at menarche in Danish data. The former study used a lower dose of metformin, but the participants were treated for a longer period. Using a more conservative approach with a change of 0.5 SD in age at menarche (equivalent to delaying menarche from 11.4 (SD 0.69) years to 11.9 years), we found that with a significance level of 5% and power of 90%, there should be 80 girls (40 in each treatment group (metformin/placebo)) included to detect a difference. However, our primary outcome is change in bone maturation (change in bone age) as an earlier surrogate measurement of age at menarche because it describes pubertal progression. No other studies have explored the effect of metformin on pubertal progression determined by changes in bone age which hinders a sample size calculation based on the primary outcome. We therefore plan to include 80 girls (metformin (*n* = 40) or placebo (*n* = 40)). We will use an intention-to-treat approach, and data from all randomized subjects will be included in the statistical analysis.

### Recruitment {15}

Eligible participants from the DAPP cohort will be approached during a visit at the outpatient clinics at the participating pediatric departments. Alternatively, potential participants from the DAPP cohort that have been identified after review of their medical notes can be contacted by letter containing a brief description of the trial and asked if they could be interested in more information about and potentially participation in the LIFE-MET trial. Informed consent from all parental authority holder(s) and assent from the children will be obtained prior to undertaking any trial-related procedures.

## Methods: assignment of interventions

### Allocation

#### Sequence generation {16a}

A block randomization list will be created for each center by our data manager (using Sealed Envelope™, www.sealedenvelope.com), at no point available for personnel enrolling participants. Blocks will consist of either 4 or 8 participants. Participants will be randomized using stratified permuted block randomization. First, participants will be divided into strata (center), and then block randomization in the four trial arms (Fig. 1) will be performed for each stratum.

#### Concealment mechanism {16b}

Random allocation to a group will be performed in REDCap upon the baseline visit, using the computer-generated allocation sequence. When clicking “randomization” in REDCap, a code and “Lifestyle intervention: YES/NO” will appear. In the locked medicine room, the investigator can find an envelope with the designated code containing tablets of either metformin or placebo.

#### Implementation {16c}

Participants and their parents will be informed, enrolled in the trial, and randomized by the recruiting investigator who will remain blinded to the allocation of metformin or placebo during the entire study period.

### Blinding

#### Who will be blinded after assignment to interventions {17a}

The pharmacy will receive the allocation list from our data manager and will label and pack the placebo and the metformin accordingly. The placebo and the metformin will be identical in appearance and will be sourced in identical packages with similar labels.

The patient, investigator, and all study and project management staff will be blinded to treatment allocation with metformin or placebo. Allocation to lifestyle intervention cannot be blinded and will thus appear as “Lifestyle intervention: YES/NO” upon allocation.

#### Circumstances under which unblinding is permissible {17b}

If an adverse event should lead to termination of the trial, or if a participant/parent wishes to withdraw from the intervention, the participant will be invited to continue to attend trial visits and to complete all trial assessments. After completion of the trial, the treatment allocation can be revealed.

In case of the need for emergency unblinding, either the data manager or a designated colleague not related to the trial can be contacted via the coordinating investigator. Only these two persons will have access to the allocation list.

## Methods: data collection, management and analysis

### Data collection and assessment of outcomes {18a}

See Fig. 2.

The primary outcome of this trial is defined as the difference in bone age (days) over 1 year determined by automatic bone age determination (BoneXpert, Visiana, Denmark [54]). An X-ray of the left hand and wrist will be performed at baseline, 6 months, and 12 months. At each center, the same version of the BoneXpert program will be employed for the determination of bone age (version 3; 2021). This method defines bone ages by Greulich & Pyle and Tanner-Whitehouse.

Secondary and exploratory outcomes:

- Clinical variables will be collected by an experienced pediatric team (pediatric endocrinologist and nurse) at baseline and at 3, 6, and 12 months: pubertal staging (Tanner stage), anthropometry (height, weight, BMI, waist-hip circumference, and ratio), systolic blood pressure (BP), and heart rate (HR).
- Body composition will be assessed by a DXA at baseline and again at 6 months and will include body fat percentage, lean mass, and gynoid/android fat distribution (g/%).
- Fasting blood samples (sex hormones, metabolic markers, and safety parameters) will be collected at baseline and again at 3 and 6 months. The maximum amount of blood drawn at each visit will be 40 ml. Safety parameters (Na, K, urea, creatinine and ALAT, bilirubin) will be analyzed immediately at the local clinical laboratories. The analyses for research purpose will be performed centrally after collection of all samples during the 2 years of inclusion. At first, the samples will be centrifuged, labeled, frozen, and stored in a research biobank at each hospital coordinated by Bio- and Genome Bank, Denmark (RBGB). The biological material will be frozen at either −20 °C or −80 °C in a locked freezer. After end of the trial and sample collection, all the serum samples in the research biobanks will be transferred to and stored in the permanent freezer facility in the Capital region (BIOSEK) so they are available for the planned analyses in the project.
- Age at menarche: Questionnaire on occurrence of menarche from the 12 month-visit and until menarche, sent biannually.
- Fitness and activity outcomes:
  o Fitness level (aerobic performance and explosive strength) will be evaluated for all participants prior to and after cessation of the intervention period using the following validated tests adapted to children: standing long jump (centimeters), a 20-m sprint (seconds), and the YYIR1C (Yo-Yo Intermittent Recovery Level 1 for Children, meters) [53].
  o Daily physical activity level will be tracked (SENS Motion® activity tracker) for 1 week prior to and after cessation of the intervention period. Activity level will be given as percentage time spent in different activity categories (i.e., rest, moderate intensity) on average.
  o Daily steps taken: Participants in the lifestyle intervention groups will be asked to wear a fitness watch every day during the 6 months intervention period and to record the number in a diary.
- Quality of life: Participants and their parents will be asked to fill out the WHO-5 Well Being Questionnaire on quality of life before and after the intervention.

### Plans to promote participant retention and complete follow-up {18b}

During the intervention period, participants will be seen with 3-month intervals. In addition, a telephone interview will be performed after 1 month of recruitment. This close contact with participants should facilitate adherence to the intervention and reduce the risk of lost to follow-up.

### Data management {19}

Data will consist of numerical data (clinical data including blood samples, calculated bone age, values from DXA, results from fitness tests, and activity level), imaging data (X-rays, DXA), and information from questionnaires. All research data will be entered in pseudo-anonymized form (patient ID generated in REDCap) and will be stored in a REDCap database. The trial database will be protected by a security code, and only registered users with a password will have access.

Blood sample results (safety parameters), X-rays, and some clinical data (e.g., anthropometry) will in addition be stored in the electronic patient journal. Electronic questionnaires will be sent directly to REDCap.

We will perform repeated monitoring of data to identify eventual missing data and to ensure the accuracy of data in order to intervene if necessary.

### Statistical methods {20a}

The statistical analyses will be performed using the program R (R version 4.3.2, 2023 or later, The R Foundation for Statistical Computing). We will apply linear mixed-effects models to assess the effect of the intervention (± metformin, ± lifestyle) on the primary endpoint: changes in bone age (days). Furthermore, we will employ mixed-effects models to assess the effect of the intervention on the secondary outcome measures: breast development, sex hormones, insulin sensitivity, metabolic markers, fitness level, and body composition within and between treatment groups as well as age at menarche. Model assumptions for both linear and logistic mixed regression models will be assessed, including, where appropriate, checks for linearity, multicollinearity, random effects normality, and residual diagnostics.

Statistical analysis will be done separately for participants who complete follow-up (per-protocol) and the intention-to-treat population. Only participants that complete the intervention (have taken a minimum of 80% of the pills and participated in a minimum of 80% of the eventual allocated lifestyle intervention) will be part of the per-protocol population. Participants must in addition complete a minimum of the baseline visit and the follow-up visit at 12 months. A *P* value of <0.05 will be considered statistically significant. Estimates of treatment effects will be presented with 95% confidence intervals.

### Methods for additional analyses {20b}

No subgroup analyses will be done.

### Definition of analysis population relating to protocol non-adherence and methods to handle missing data {20c}

The intention-to-treat analysis will include all randomized subjects, ignoring non-compliance, protocol deviations, and withdrawal for any reason. Every effort will be made to obtain missing data, but missing data will be handled appropriately to minimize biases. We will explore the reasons for missing data and assess whether it is ignorable, or if it depends on other variables. Possible techniques to handle this could be imputation (e.g., mean imputation, multiple imputation).

## Methods: monitoring

### Data monitoring committee {21a}

A study steering group consisting of the principal investigator and a Data Monitoring Committee (DMC) with an independent chair has been constituted with the purpose of assessing the progress and safety data of the LIFE-MET trial. The DMC will review the safety data every 6 months during the trial and report to the chief investigator and the sponsor. Based on its review, the DMC provides the sponsor with recommendations regarding trial modification, continuation, or termination. These reports will be included in the annual reports to the ethics committee. Further information can be found in CTIS.

### Interim analyses {21b}

No interim analysis will be performed. Statistical analysis will be done once the trial is completed.

### Harms {22}

Information on any potential adverse event or reaction (AE/AR) including its nature and severity will be collected at the local investigating site on clinical visits at 3 and 6 months and by telephone after 1 month of treatment. The principal investigator at each trial site will be responsible for performing supplemental measurements and/or evaluations as individually and medically indicated in case of adverse reactions. This could include physical examination and blood sampling, and if indicated, admittance to the local department of pediatrics. Participants will be followed up until resolution or stabilization, or until the event has been shown to be unrelated to the drug. Serious adverse events and serious adverse reactions should be reported to the sponsor immediately. AEs and ARs will be documented in the electronic case report form (eCRF) and in the “Summary of results” report that will be uploaded in CTIS within 1 year after completion of the trial. In addition, an annual safety report including a report on the safety of metformin will be submitted through CTIS.

### Auditing {23}

The trial will be conducted in compliance with the principles of Good Clinical Practice (GCP) and will be audited by the Danish GCP Units. Audits will be performed before the initiation of the trial, after the enrollment of a few participants, and continuously thereafter during the conduct of the trial as well as after the trial has ended. This process will be independent from both the sponsor and the local investigators.

### Protocol amendments {25}

Important protocol modifications must be approved by the CTIS and will be communicated to trial investigators.

### Consent or assent {26a}

Informed consent from all parental authority holder(s) and assent from the children will be obtained by the primary investigator prior to undertaking any trial-related procedures.

### Additional consent provisions for collection and use of participant data and biological specimens {26b} and plans for collection and storage of biological specimens for genetic or molecular use in this/future trials {33}

The period of storage of serum samples is 10 years from the date when the samples are transferred to the research biobank which is closed on the 31 st of October 2036. After this date, the remaining serum will be transferred to a biobank available for future research projects. Storage in the research biobank and in the biobank for future studies is performed according to and after obtaining informed signed consent from the parents of all subjects regarding the storage of the biological material.

### Confidentiality {27}

All research data will be pseudo-anonymized using a unique patient identifier. Collected data will be stored in a common REDCap database in compliance with relevant data protection regulations.

### Ancillary and post-trial care {30}

Not applicable. No such provisions have been made.

### Dissemination policy

#### Communication of trial results {31a}

All results, positive, negative, or inconclusive, will be disseminated at international conferences or as peer-reviewed scientific journal articles in pediatric and epidemiological-oriented journals of the highest possible impact. We will ensure relevant press releases of our new findings and updated data on publicly available websites including www.tidligpubertet.dk. The trial has been registered at www.clinicaltrials.gov as part of the DAPP study (Clinical Trial number NCT05957991). Results will be submitted to CTIS within 1 year of the end of the trial.

#### Reproducible research {31c}

The full protocol is publicly available in CTIS. The statistical code can be obtained from the authors. Data cannot be fully anonymized at the participant level (will be pseudo-anonymized) and will therefore not be publicly available.

## Discussion

Age of pubertal development has been declining for decades, resulting in increased attention on the accompanying risks of adverse health outcomes and psychosocial challenges for the affected girls [15]. In addition, early puberty in girls is often related to overweight, a condition affecting approximately one third of European children today [26]. The precise mechanisms linking early puberty and overweight remain to be fully clarified, but several hypotheses have been suggested. Currently, there is no approved treatment for girls entering puberty after 8 years of age. Even for girls who enter puberty before age 8 years, most of whom have non-pathological precocious puberty, treatment options are invasive and do not address the underlying mechanisms. New strategies not only for treatment but also for the prevention of both overweight and early puberty are obviously needed.

The LIFE-MET trial is a randomized controlled trial that uniquely combines a lifestyle intervention, utilizing online virtual reality training, with a pharmacological intervention involving metformin and/or placebo, with the objective of delaying further pubertal advancement.

Several studies support the hypotheses that both physical activity, weight loss, and metformin therapy will improve insulin sensitivity and thereby contribute to delaying pubertal onset or further pubertal progression in girls with overweight [38–41].

Insulin resistance and the resultant hyperinsulinemia are thought to play a key role linking adiposity and early puberty due to increased levels of androgens from both the adrenal glands and ovaries and decreased concentration of SHBG, resulting in an increase in bioavailability of sex steroids. The increased levels of sex steroids may trigger stimulation of the HPG axis and lead to central precocious puberty or directly promote precocious adrenarche, pubarche, or precocious thelarche [24, 29]. High concentrations of insulin in women and rodents have also been associated with increased LH secretion [55, 56].

Another potential explanation includes the role of aromatase from adipose tissue, converting androgens into estrogens and thereby facilitating both isolated breast development and also direct stimulation of the HPG axis [24, 29].

Furthermore, adipocytes possess endocrine functions, and the relationship between obesity and central precocious puberty (CPP) in girls involves metabolic hormones and neuropeptides, in particular the adipokine leptin and the neuropeptide kisspeptin, which both interact with and modulate the hypothalamic circuits regulating reproductive functions [57]. Leptin, an indicator of subcutaneous fat stores and thus elevated in individuals with obesity, rises gradually during the peripubertal period and signals when energy stores are sufficient for initiation of puberty, but it cannot alone trigger the onset of puberty [24, 29, 58]. Kisspeptin stimulates the secretion of gonadotropin-releasing hormone (GnRH) and has been described as a *gatekeeper* of pubertal onset that is sensitive to the metabolic state, although findings indicate that Kiss1 neurons do not serve as the primary pathway through which metabolic status influences the HPG axis [59, 60]. The central melanocortin pathway, which integrates signals for energy status from both leptin and insulin, has also been shown to play a role in the initiation of puberty [61]. A recent study suggested that melanocortin-3 receptor (MC3R) signaling plays an important role in the regulation of caloric allocation toward growth, the development of lean body mass, and the timing of sexual maturation [62]. The expression of the melanocortin-3 receptor (MC3R) is elevated in hypothalamic neurons that control growth and reproduction (GHRH and kisspeptin-neurokinin B-dynorphin (KNDy) neurons) [63] and in mice, the expression of this receptor increased postnatally in a manner suggesting a role in the regulation of pubertal development [62]. In the same study, MC3R loss-of-function (LoF) mutations identified in humans resulted in delayed puberty. Brain ceramides, known for their role in glucose homeostasis, may also contribute to the link between childhood obesity and control of female puberty, as central inhibition of ceramide synthesis was found to counteract the effects of kisspeptin and delay the onset of puberty in a recent animal study [64, 65].

The endocrine actions of adipose tissue could explain the effect of weight loss or changes in body composition on pubertal development, assuming that a reduction in fat mass leads to reduced endocrine activity of the adipose tissue and hence a delay in pubertal onset or further progression. Supporting this, a recent Chinese study demonstrated that a high fat mass is more closely linked to early pubertal onset than BMI [66].

To augment physical activity levels and assist in weight management among children, active video games may serve as a supplementary approach; however, existing evidence indicates that their effects are limited and insufficient for standalone intervention [44, 50, 67]. Evidence regarding online, VR-training in children is sparse, and more knowledge and experience are needed before considering implementing this strategy on a larger scale. We aim to investigate the potential of a fitness application that facilitates interactions among children, peers, and coaches within a dedicated online virtual reality environment. We hypothesize that this innovative format could enhance motivation, thereby improving adherence to lifestyle modifications that promote a more active daily life.

The primary strength of our trial is the randomized, placebo-controlled design that should ensure comparable groups at baseline and hence avoid selection bias. In addition, the trial will be monitored by the Good Clinical Practice unit, and deviations from the protocol must be reported, minimizing selective outcome reporting and bias arising from deviations from planned interventions. Obvious limitations are the limited sample size and the duration of the trial which could challenge the power to detect differences between groups.

In conclusion, this RCT will test the effects of lifestyle and pharmacological interventions on the rate of pubertal progression, with potential beneficial effects also on body composition. A healthier body composition may have favorable effects on long-term co-morbidities related to early puberty. We hypothesize that these positive changes will be mediated by enhanced insulin sensitivity, a reduction in adipose tissue mass, and, as a result, reduced endocrine activity of adipose tissue.

## Trial status

Recruiting. Inclusion of first patient on March 20, 2025. Expected end of recruitment: February 20, 2027.

Protocol version: 1.3, 17/10/2024.

## Data Availability

A clinical trial agreement has been made defining that each trial site is responsible for and will have access to the data collected at that site. The coordinating investigators, statisticians, and researchers at the sponsor site will have access to the final data set after the termination of the trial (last patient’s last visit).

## Abbreviations

AE/AR: Adverse event/adverse reaction
AVGs: Active video games
AVRGs: Active virtual reality games
BMI: Body mass index
BP: Blood pressure
CPP: Central precocious puberty
CTIS: Clinical Trial Information System
DAPP: DAnish Precocious Puberty study
DXA: Dual-energy X-ray absorptiometry, DEXA-scanning
eCRF: Electronic case report form
EDC: Endocrine disrupting chemical(s)
GCP: Good Clinical Practice
GHRH: Growth hormone-releasing hormone
GnRH: Gonadotropin-releasing hormone
HOMA-IR: Homeostatic Model Assessment for Insulin Resistance
HPG: Hypothalamic-pituitary-gonadal (axis)
HR: Heart rate
KNDy: Kisspeptin-neurokinin B-dynorphin (neurons)
LoF: Loss of function mutation
MC3R: Melanocortin-3 receptor
PA: Physical activity
PT: Premature thelarche
PI: Principal investigator
QoL: Quality of life
RBGB: Regional Bio- and Genome Bank, Denmark
RCT: Randomized controlled trial
SGA: Small for gestational age
SHBG: Sex hormone binding globulin
VR: Virtual reality
WP: Work package
